# Percutaneous Axillary Left Ventricular Support as a Lifesaving Measure for Peripartum Cardiomyopathy Shock: A Case Report

**DOI:** 10.7759/cureus.44224

**Published:** 2023-08-27

**Authors:** Smruti Desai, Shriya Sharma, Smit Paghdar, Jose Ruiz, Rohan Goswami

**Affiliations:** 1 Division of Heart Failure and Transplant, Mayo Clinic, Jacksonville, USA; 2 Internal Medicine, Surat Municipal Institute of Medical Education and Research (SMIMER), Surat, IND

**Keywords:** peripartum, heart transplant, bridge to transplant, mechanical circulatory support, impella cp, transplantation, heart failure

## Abstract

Peripartum cardiomyopathy (PPCM) affects women at the end of pregnancy or after delivery. Symptoms overlapping with pregnancy decrease the diagnostic yield of PPCM and can increase the rate of maternal mortality. As clinicians manage high-risk patients, it is crucial to understand the variable maternal physiology both during and after childbirth. Effective management of high-risk patients necessitates a comprehensive understanding of the variable maternal physiology during and after childbirth. The importance of prompt intervention with Impella CP (Cardiac Power) to treat acute cardiogenic shock stemming from PPCM after cesarean should be considered. Clinical outcomes can be improved by emphasizing the need for timely intervention and incorporating a comprehensive understanding of maternal physiology. The authors present a case of Impella CP use in PPCM shock as a means for emergent support after cesarean.

## Introduction

Peripartum cardiomyopathy (PPCM) is a form of heart failure that primarily affects young women during the later stages of pregnancy or in the months following childbirth. Most PPCM cases are identified after delivery, typically within the first month. It is more commonly observed in African American women and those with advanced maternal age, hypertensive disorders during pregnancy, or multiple pregnancies. The symptoms of heart failure in PPCM often resemble those experienced during a normal pregnancy, contributing to frequent delays in diagnosis.

PPCM is a pregnancy-related new-onset heart failure affecting women toward the end of pregnancy or, most commonly, in the week after delivery [1]. According to the American Heart Association, PPCM affects 1000-1300 women annually in the United States [2]. This prevalence is likely underestimated and represents a lack of awareness of this condition and probable missed diagnosis due to confounding symptoms. Difficulty in diagnosing PPCM results from the symptoms of heart failure during pregnancy mimicking the normal physiological changes during the late stages of pregnancy [3]. The prevalence of pre-eclampsia or other hypertensive disorders has a strong predisposition to PPCM, and their symptoms of shortness of breath and peripheral edema can also contribute to a delay in diagnosis [4]. The diagnostic criteria of PPCM include 1) evidence of heart failure within the last month of pregnancy or five months after delivery, 2) no other identifiable causes of heart failure with reduced ejection fraction (EF), 3) no apparent heart disease before the last month before delivery, and 4) echocardiographic evidence showing decreased left ventricular (LV) systolic function [1].

We present a case of Impella CP (Cardiac Power) use in PPCM shock as a means for emergent support after cesarean section [1]. Minimally invasive temporary heart pumps, such as the Impella CP - a trans-axial pump placed through the femoral or axillary artery and into the LV cavity after crossing the aortic valve are utilized to improve cardiac output with an increase in flow up to 2.5 L/min - have been developed and effectively used to enhance outcomes in cases where immediate but not necessarily long-term support is required.

## Case presentation

**Figure 1:**
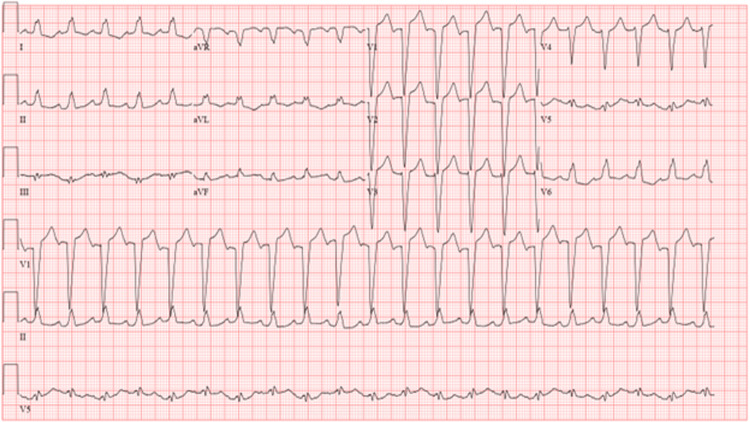
Baseline screening electrocardiogram demonstrating sinus tachycardia with a left bundle branch block with strain pattern

A 24-year-old female G1P0 at 36 ½ weeks gestation known to have hypertension, gestational diabetes, and preeclampsia presented with a one-week history of shortness of breath, chest pain, orthopnea, increased lower extremity edema, palpitations, and tachycardia. Upon arrival, an electrocardiogram was done and showed sinus tachycardia with a left bundle branch block (Figure 1).

Transthoracic echocardiogram revealed left ventricular (LV) dilatation with an LV end-diastolic diameter of 7 cm with EF estimated to be 20% to 25%. The right ventricle (RV) was mildly enlarged and moderately hypokinetic with an RV pressure of 66 mmHg. Subsequently, she developed significant hypoxemia, and a computed tomography angiography was done to rule out pulmonary embolism or aortic dissection, revealing alveolar changes and an enlarged cardiac silhouette. These findings prompted a cesarean section, delivering a healthy baby.

**Figure 2:**
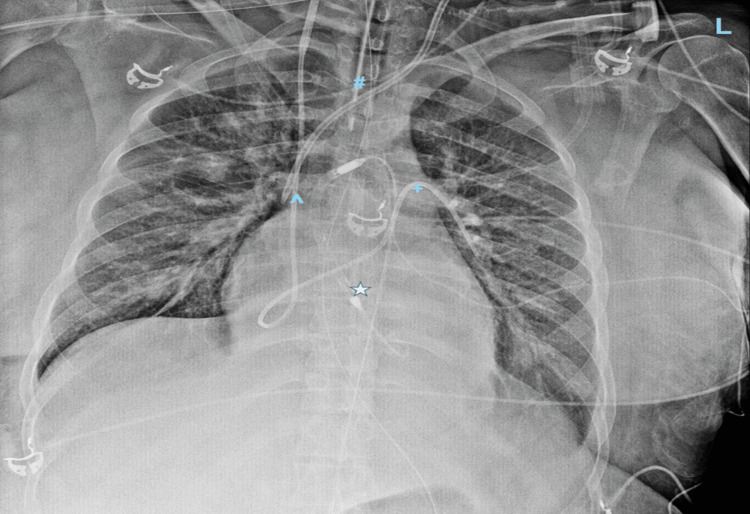
Antero-posterior chest x-ray demonstrating Impella CP device placement in LV cavity *Impella CP via femoral approach, ^#^endotracheal tube, ^central venous line, and ^+^pulmonary artery catheter with an enlarged cardiac silhouette and pulmonary congestion. LV, left ventricular

After her cesarean section, she had episodic supraventricular tachycardias that were unresponsive to adenosine and required initiation of intravenous amiodarone. Due to further decompensation, requiring a high-flow nasal cannula, she underwent evaluation for acute mechanical circulatory support while on norepinephrine 0.02 mcg/kg/min and milrinone 0.25 mcg/kg/min. Ultimately requiring intubation, she was taken to the cardiac catheterization lab for femoral Impella CP placement (Figure 2).

Pre-impella pulmonary artery (PA) catheterization revealed right atrial pressure (RA) of 18 mmHg, PA pressure of 45/21 (29) mmHg, pulmonary capillary wedge pressure (PCWP) of 37 mmHg, left ventricular end-diastolic pressure (LVEDP) of 32 mmHg, cardiac output of 2.5 L/min, and cardiac index 1.37 L/min/m^2^. Following the implantation of the Impella, her urine output improved, and she was partially weaned from inotropic support but remained on milrinone 0.25 mcg/kg/min. With favorable lactate dehydrogenase trends and successful fluid removal, her cardiac function showed improvement, and her Impella was removed on postoperative day five. However, the patient could not tolerate complete wean from inotropic support with milrinone 0.25mcg/kg/min as assessed by venous blood gas, Fick cardiac index calculation, and patient symptoms. She was discharged on home milrinone with close follow-up after a two-week hospitalization. Her outpatient plan was to optimize guideline-directed medical therapy (GDMT) and attempt to wean inotropic support with the hope of myocardial recovery.

## Discussion

Screening and management of PPCM

Given the broad differential for shortness of breath in gravid females, our case provides insight into early screening and diagnosis for PPCM with a surface echocardiogram. Upon suspicion of PPCM, echocardiography should be performed, as the left ventricular ejection fraction (LVEF) is typically <45% when establishing the diagnosis [5]. This can detect high-risk patients before they progress to severe forms of the disease. The management is tailored to the severity of each individual patient, which could range from starting GDMT, initiating inotropic support, or considering mechanical support in patients with florid cardiogenic shock. There are few other options in the acute setting of shock. Pre-delivery screening is crucial in identifying patients with risk for PPCM but is limited in their sensitivity, such as hypertension, proteinuria, or symptoms of volume overload. Recovery after PPCM diagnosis depends on the severity and is limited. Some case series reports of around 37% have been published [6]. Utilizing the team approach with cardio-obstetric specialists, intensivists, and heart failure cardiologists may highlight the need for planned support before delivery in higher-risk mothers.​

As providers manage high-risk patients, it is crucial to understand the variable maternal physiology both during and after childbirth. In this case, we describe treatment options that are safe and effective yet often overlooked. Supporting post-partum mothers with mechanical support and allowing time for organ recovery and optimization of deranged hemodynamics, vascular tone, and hormone cascades during the critical period after delivery is key in their management. The importance of prompt intervention with Impella CP to treat acute cardiogenic shock stemming from PPCM after cesarean should be considered. 

## Conclusions

This case explores the utility of early intervention after cesarean and support with Impella CP placement as a bridge to recovery from PPCM. Salvage therapy with the Impella CP as a bridge to recovery, transplant, or durable ventricular assist device can be a viable option in the subset of patients with acute shock. By providing hemodynamic stabilization and LV unloading, Impella CP plays a crucial role in managing severe myocardial insult and cardiogenic shock associated with PPCM. Physicians must be aware of the challenges in diagnosing PPCM promptly due to overlapping symptoms with normal pregnancy and the postpartum period. Our article highlights the importance of Impella CP in the management of acute cardiogenic shock arising from PPCM after cesarean.
